# Multimodal imaging observation of primary vitreous cysts

**DOI:** 10.1186/s12886-024-03482-x

**Published:** 2024-05-21

**Authors:** Siduo Lu, Ning Cai, Di Yang

**Affiliations:** Department of Ophthalmology, The First Affiliated Hospital of Kunming Medical University, Kunming, Yunnan Province China

**Keywords:** Vitreous cyst, Idiopathic cyst, Multimodal imaging, Case report

## Abstract

**Background:**

Primary vitreous cyst is a clinical variant delineated by the existence of a vesicle within the vitreous cavity from birth. This particular disease tends to be uncommon, and the underlying mechanisms contributing to its pathogenesis remain obscure.

**Case presentation:**

A 37-year-old male patient manifested blurry vision and floaters in his right eye, a symptomology first noticed three months prior. Upon slit-lamp examination, a pigmented, round, 1 papilla diameter-sized mass was discerned floating in the vitreous. A meticulous examination of the floaters was conducted using an array of multimodal imaging techniques. Other potential conditions, including cysticercosis, toxoplasmosis, and tumors, were conclusively excluded through comprehensive diagnostic tests such as blood examinations, liver ultrasound, and cranial magnetic resonance imaging (MRI), resulting in the diagnosis of a primary vitreous cyst. The patient did not report any other discomforts and did not receive any subsequent interventions or treatments.

**Conclusion:**

We furnish an exhaustive case report of a patient diagnosed with a primary vitreous cyst. The incorporation of multimodal images in the characterization of the disease anticipates facilitating an enriched comprehension by medical practitioners.

## Background

Vitreous cysts represent an exceedingly rare category of ocular malformation and are distinguished as either congenital or acquired, pigmented or nonpigmented, and predominantly asymptomatic [1]. These cysts exhibit variation in both mobility, ranging from free-floating to fixed, and morphology, assuming spherical, oval, or lobulated configurations. The demographic incidence of vitreous cysts spans between 5 and 68 years of age [2]. While the majority of vitreous cysts remain benign, manifesting neither symptoms nor requiring intervention, their dimensions may vary from 0.15 mm to 12 mm [3]. In instances where cysts instigate visual disruptions, medical interventions such as argon or Nd-YAG laser photocystotomy, and pars plana vitrectomy may be necessitated [4, 5]. In the context of this study, we extend a comprehensive elucidation of a patient’s case of primary vitreous cyst employing multimodal imaging techniques, an approach that has not been previously documented in the extant literature.

## Case presentation

**Fig. 1:**
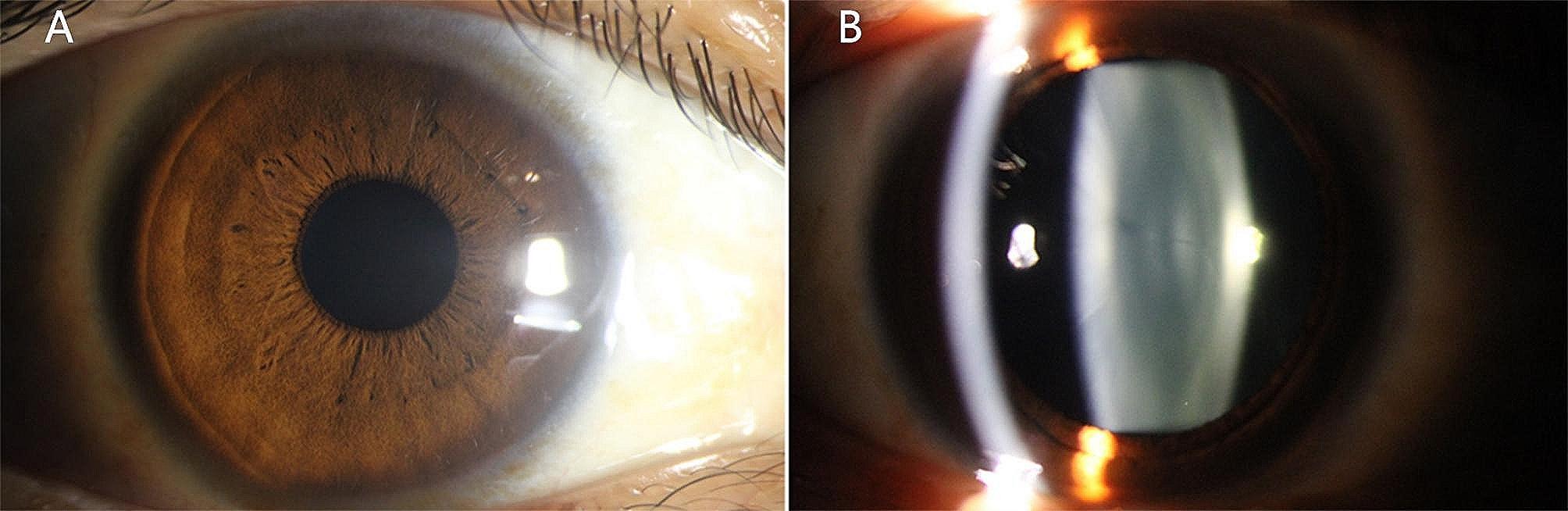
Slit lamp photography revealed a normal anterior segment. (A) Direct diffuse illumination. (B) Direct optical section illumination.

**Fig. 2:**
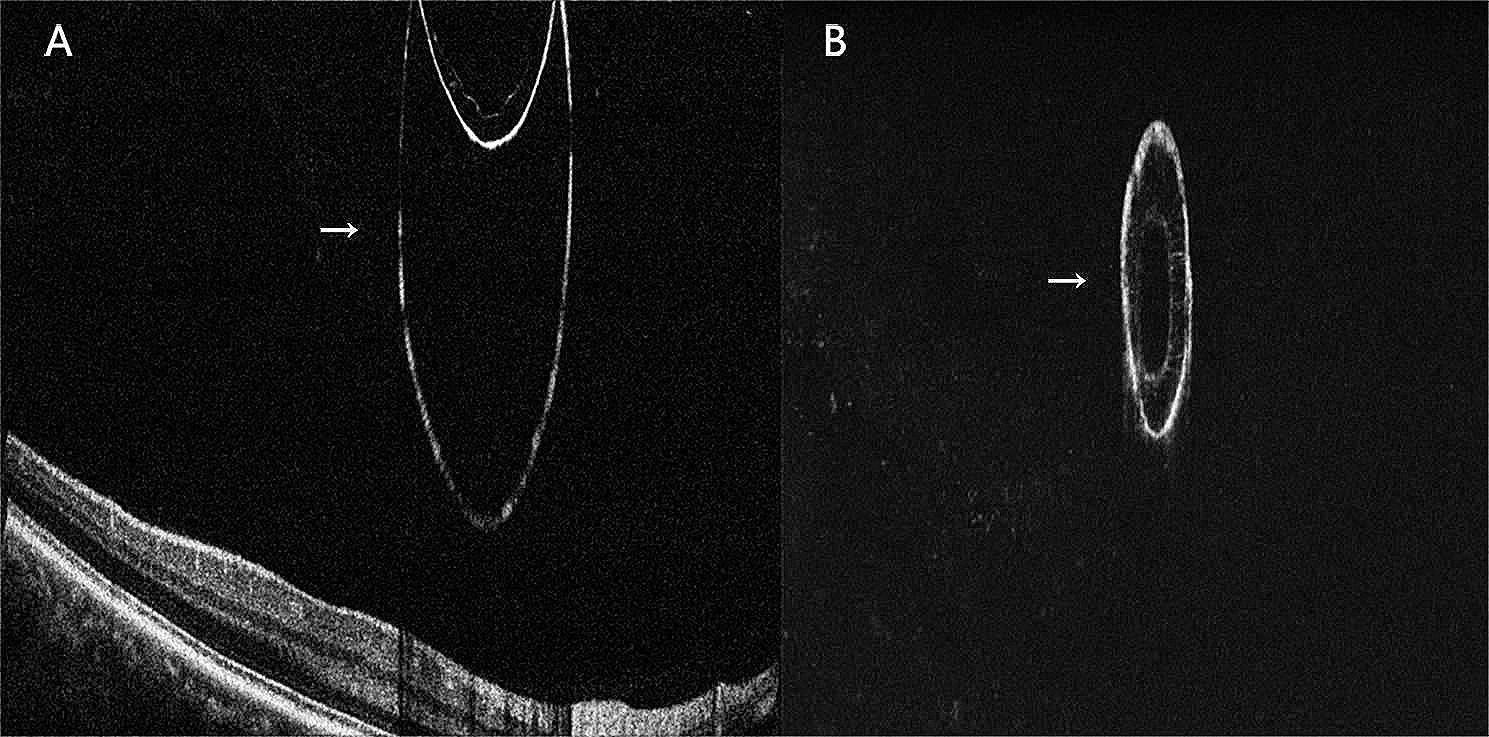
OCT imaging shows the internal structure of the vitreous cyst. Arrows indicate the cyst. (A) Relative position between vitreous cyst and retina. (B) Characteristics of the vitreous cyst.

**Fig. 3:**
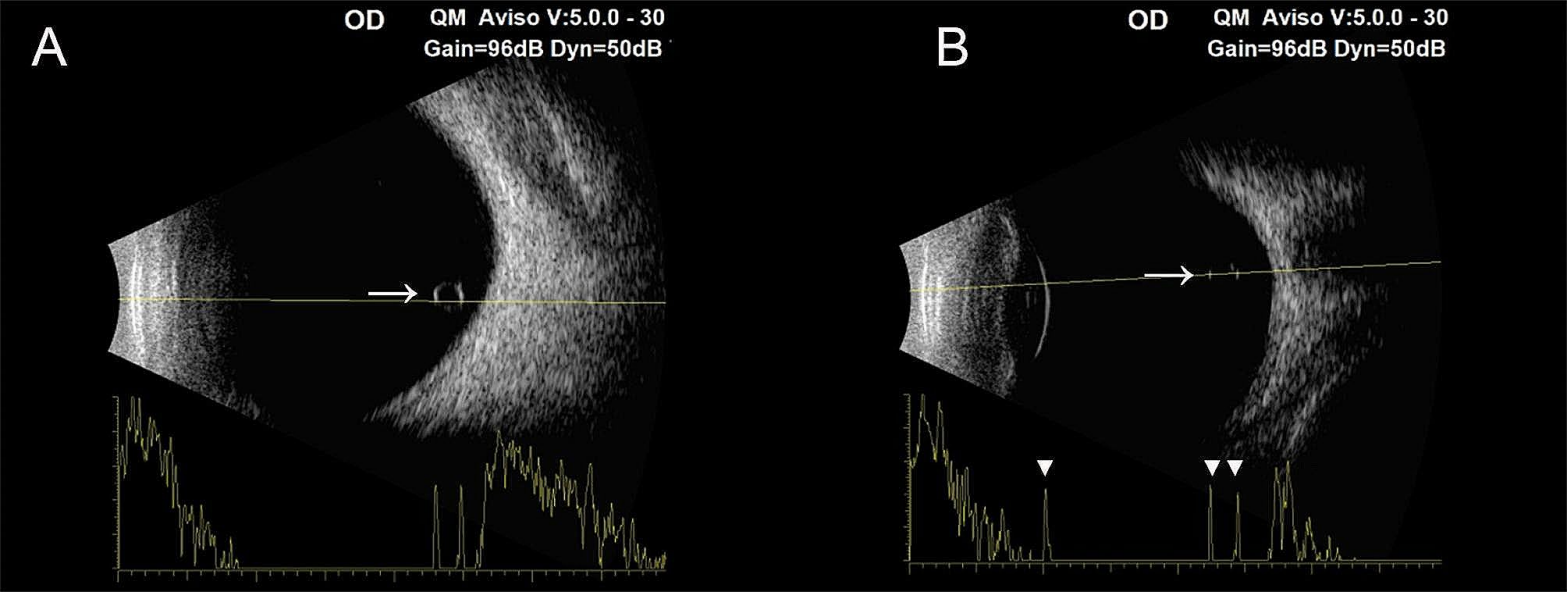
Ocular B-scan ultrasonography of the patient’s right eye. Arrows indicate the cyst wall (A), and triangles indicate the echo intensity of the cyst wall and the lens posterior capsule (B).

**Fig. 4:**
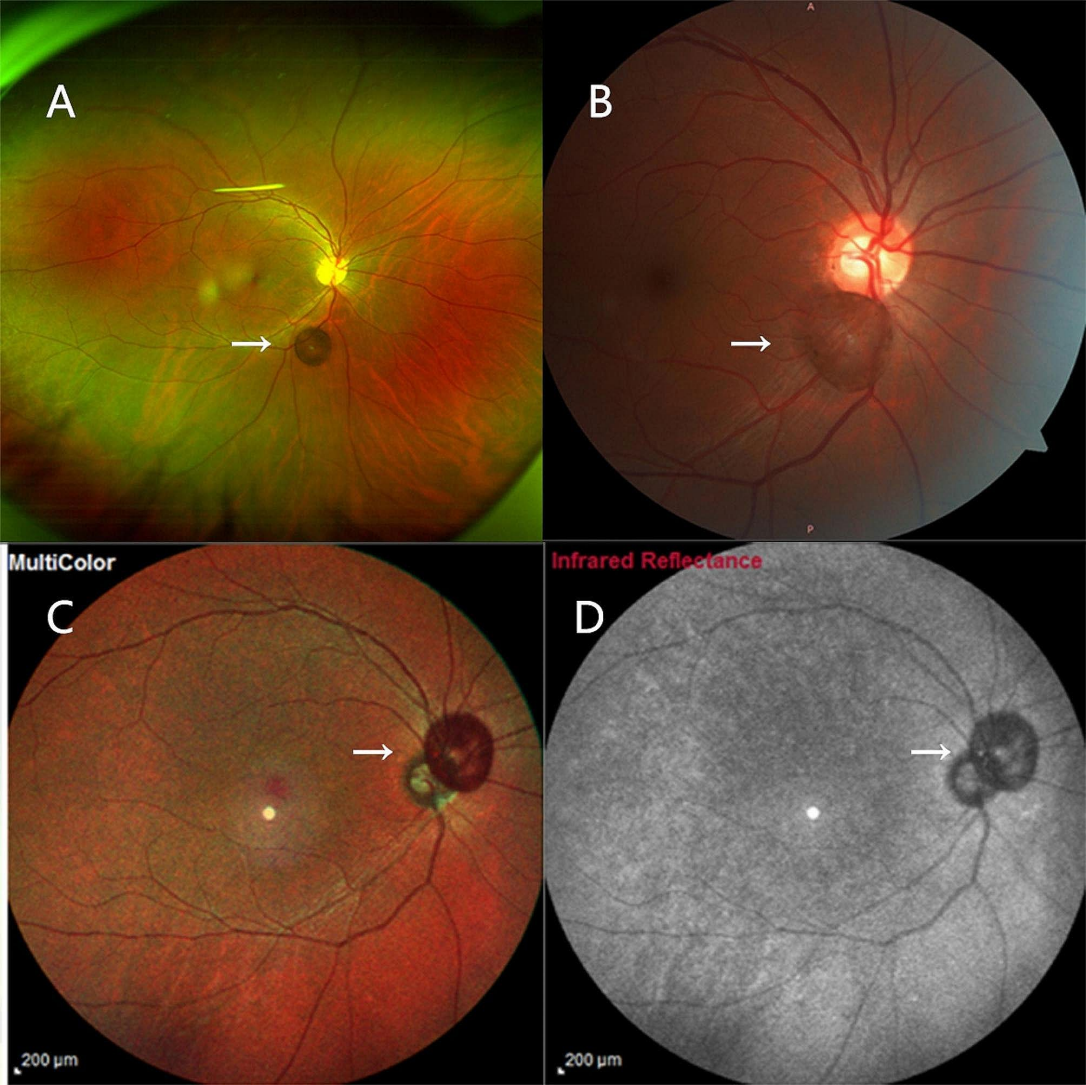
Scanning laser ophthalmoscopy (A), fundus photography (B), multicolor imaging (C), and Infrared Reflectance (D) of the patient’s right eye. Arrows indicate the cyst.

A 37-year-old Chinese male disclosed the existence of a round-shaped floater in his right eye, first observed approximately 3 months prior, causing recurrent visual perturbations. The patient’s medical background is devoid of any antecedent ocular or systemic maladies. The patient denied any history of ocular trauma or surgery. A measurement of visual acuity with optimal correction revealed a 20/20 score in both eyes. The slit lamp examination conducted detected a free-floating pigmented vitreous cyst within the vitreous cavity of the right eye. In addition to the cyst’s presence, a meticulous bilateral ocular examination elucidated no discernible aberrations. Moreover, no manifestations of peripheral retina degeneration were detected in either eye. Subsequent color photography of the anterior segment did not uncover any conspicuous abnormalities (Fig. 1). A detailed evaluation of the vitreous cyst confirmed its cystic morphology without the appearance of scolex by using Optic Coherence Tomography (OCT). OCT imaging demonstrated a thin, hyperreflective cyst wall, while the interior of the cyst consisted of numerous hyperreflective septa, partitioning the lumen into a multi-lobular configuration (Fig. 2). B-scan ultrasound examination delineated a circumscribed region of augmented echogenicity within the vitreous body adjacent to the retina. No linkage between the vitreous cyst and other ocular structures was observed on the B-scan ultrasound. Furthermore, the echo intensity of the capsule wall was congruent with that of the lens posterior capsule (Fig. 3). Supplementary fundus examinations were pursued to further explicate the cyst’s characteristics (Fig. 4). Of those, Infrared Reflectance imaging shows high reflectance in specific areas, thereby emphasizing the existence of melanosomes.

A series of auxiliary tests were meticulously conducted to preclude possible alternative etiologies. The MRI of the orbit and skull did not reveal any marked abnormalities. The abdominal ultrasound examination corroborated a normal liver size and morphology and uniform density. Complete blood cell count, erythrocyte sedimentation rate, and C-reactive protein levels were within normal range. Serological assays for antibodies against herpes simplex virus, rubella virus, Toxoplasma gondii, Echinococcus, cysticercoids and Toxocara canis species returned negative results, and stool examination for ova and cysts were also negative. Through a synthesis of the patient’s medical history, comprehensive ophthalmic examinations, and supportive diagnostic assays, conditions such as parasitic infection were excluded, culminating in the diagnosis of an primary pigmented vitreous cyst. Currently, the patient has been followed up for 6 months and retains satisfactory visual acuity without additional discomfort. To date, no specialized treatments have been instituted, and periodic follow-ups for 2 years will be continued to vigilantly observe any alterations in the condition.

## Discussion

Vitreous cysts can be either congenital or acquired, the mean age of patients ranges from 5 to 68 years [1]. Congenital vitreous cysts, cystic formations extant within the vitreous cavity from birth, present a complex and intriguing clinical scenario [6]. These entities are relatively rare and frequently remain undetected until later stages of life. This delayed detection is partly due to infants’ inability to articulate visual impairment, coupled with the cysts’ potential location outside the visual axis [7]. As exemplified in the cases we report, the discovery of the disease predominantly occurs in middle age. Acquired vitreous cysts are often secondary to trauma, inflammation, uveitis, and retinal detachment procedures, among others [1, 8].

The precise etiological mechanisms underlying primary vitreous cysts continue to elude understanding, although three theories have been proposed to delineate their origin: (1) Histological examinations and ultrastructural analyses have disclosed walls constituted of pigmented or non-pigmented epithelial cells, indicative of potential formation from the detachment of iris or ciliary epithelium during embryonic maturation, followed by inclusion in the vitreous. (2) The derivation from the retinal pigment epithelium. (3) The origination from the hyaloid artery or residual primary vitreous mesenchymal tissue [3, 8–10]. Jing Lu et al. performed a spontaneous fluorescence examination in a case of idiopathic vitreous cyst with an intact retina, revealing no lipofuscin presence within the cyst. This engendered a hypothesis regarding the cyst’s potential genesis from the ciliary rather than retinal pigment epithelium, a proposition worth rigorous investigation despite our reported case not corroborating this notion [11]. Additionally, studies highlighting anatomopathological analyses have unveiled a pigmented vitreous cyst, possibly of congenital inception, and described it as cystic choristoma originating from the primitive hyaloid system [10, 12]. Jones’s observation suggests that pigmented cysts within the vitreous are likely to arise from the iris region, contrasting non-pigmented cysts, which may emanate from remnants of the hyaloid system, however [13]. In our case, we speculate that the origin of the vesicle might be the ectoderm, as evidenced by its transparency and pigmented wall. Conversely, if it was derived from the mesoderm, it would likely exhibit opacity and contain residual blood vessels, indicating a source from the retinal artery.

Children diagnosed with congenital vitreous cysts are often incapable of expressing visual disturbances and are predominantly identified during routine assessments for squint or amblyopia. Conversely, adults commonly seek clinical intervention due to persistent floaters or recurrent visual deterioration. Though typically unilateral, the condition might manifest bilaterally [3]. Our case resonates with the archetypal presentation of vitreous cysts further enriching the clinical literature.

Anterior segment and fundus examinations usually yield normal findings, whereas the vitreous may demonstrate diverse degrees of liquefaction. Within this domain, semi-transparent, bubble-like configurations of either spherical or elliptical geometries, ranging from 2 to 12 mm in diameter, are discernible. These may evince subtle motility or float with eye movement, and the retina may display cystic protrusions. Such cysts often permit the visualization of underlying retinal vessels due to their semi-transparent nature and may harbor pigmentation. Distinctive semi-transparent strands, either free-floating or optic disc-attached, may be present at one end of the cysts [7]. The extraordinary documentation of an idiopathic pigmented vitreous cyst, devoid of continuous hyaloid artery, no signs of traction or attachment associated with ocular structure, or any changes at the macula by Sethi and colleagues, finds comprehensive affirmation in the OCT results of our case [14]. Furthermore, our OCT examination yielded intriguing results, as it unveiled the intricate internal composition of the cyst, characterized by numerous membranes exhibiting high reflectivity and dividing the lumen into lobular structures (Fig. 2). Notably, this represents the second instance in which a complete internal structure of the cyst has been discovered through OCT images [15], and we hypothesize that these membranes may be formed by the residual epithelial cells. Beyond this, we also found that the echo of the cyst wall is highly consistent with that of the posterior lens capsule observed in the B-ultrasound examination (Fig. 3B). This finding provides valuable insights into the potential origin of the cyst, suggesting a possible association between lens epithelial cells and cyst formation, albeit without a precise understanding of the underlying mechanism at present. Consequently, further investigation into this phenomenon remains imperative.

The diagnosis of primary vitreous cysts becomes unequivocal upon the exclusion of trauma, surgery, infectious, and inflammatory diseases, and the identification of semi-transparent or pigmented cystic structures within the vitreous cavity. It is imperative to meticulously differentiate these from vitreous cysticercosis and secondary cysts [1]. The differential diagnostic process for the case we delineate did not present substantial challenges. Vitreous cysticercosis can be swiftly ruled out, as its typical presentation includes a turbid cyst with golden-yellow reflections at the edge when illuminated, in addition to a visible scolex and suckers within the cyst, features entirely incongruent with our case. Secondary vitreous cysts, including metastatic tumors within the vitreous, traumatic vitreous cysts or cysts resulting from retinal inflammation or degenerative modifications, are effortlessly excluded in our case based on the patient’s comprehensive medical history, and manifestations of the primary disease.

Conservative management, as the literature delineates, has been the preferred recourse in reported instances [16]. Should the cysts be situated outside the visual axis without any discernible impact on vision, therapeutic intervention is rendered unnecessary. Taking all factors into consideration, we consider that the patient’s current symptoms are mild, and surgery may not be cost-effective. The patient opted for conservative management. However, significant symptomatology compels intervention, providing several therapeutic alternatives. The first encompasses laser disruption. Awan suggested argon laser photocystectomy as a safe and effective method for treating vitreous cysts [17]. However, Nork and Milechia reported an unintentional retinal burn that occurred during argon green laser cystectomy [10]. Later, Ruby etc. described Nd: YAG laser cystectomy to destroy such cysts and have remained free of recurrences [5]. Whereas, this technique is also not without complications, such as resulting in an iatrogenic cataract formation [4]. Recently, the introduction of 577 nm micropulse diode laser technology has exhibited its efficacy and safety. Photocystotomy utilizing a micropulse diode laser at multiple points presents a secure method for treating symptomatic cysts, thereby circumventing the potential hazards associated with conventional argon or Nd: YAG laser cystotomy [18]. The second pertains to vitrectomy via pars plana accompanied by cyst excision. Although it is not recommended for individuals who are asymptomatic or have mild symptoms, patients with severe visual impairment necessitate surgical removal of the vitreous body and cloudy cysts to regain their vision [4]. The third option amalgamates laser disruption with pars plana vitrectomy, which was advised in the context of pigmented cysts in severely disabled patients, as the concentration of pigment may increase with laser photocoagulation [10]. To summarize, the treatment paradigm for vitreous cysts remains multifaceted, contingent upon patient preferences, symptomatology, the degree of visual impairment, and the specific characteristics and localization of the cyst. It falls upon the medical team to judiciously navigate the therapeutic pathways to concurrently minimize patient risk and amplify benefits in a cost-efficient manner.

## Conclusion

This study presents a comprehensive case report of a patient diagnosed with primary vitreous cyst, accompanied by a literature review, emphasizing the rarity of this condition. The primary objective of this report is to enhance physicians’ clinical knowledge and awareness regarding this rare disease.

## Data Availability

The datasets used and/or analyzed in the course of the current study are available from the corresponding author on reasonable request.

## Abbreviations

OCT: Optic Coherence Tomography
MRI: magnetic resonance imaging
